# Preserving equipoise and performing randomised trials for COVID-19 social distancing interventions

**DOI:** 10.1017/S2045796020000992

**Published:** 2020-10-28

**Authors:** Ioana Alina Cristea, Florian Naudet, John P. A. Ioannidis

**Affiliations:** 1 Department of Brain and Behavioral Sciences, University of Pavia, Pavia, Italy; 2 University Rennes, CHU Rennes, Inserm, CIC 1414 (Centre d'Investigation Clinique de Rennes), F-35000, Rennes, France; 3 Departments of Medicine, of Epidemiology and Population Health of Biomedical Data Science, and of Statistics, and Meta-Research Innovation Center at Stanford (METRICS), Stanford University, Stanford, California, USA

**Keywords:** Randomisation, randomised-controlled trials, research design and methods, statistics

## Abstract

In the coronavirus disease 2019 (COVID-19) pandemic, a large number of non-pharmaceutical measures that pertain to the wider group of social distancing interventions (e.g. public gathering bans, closures of schools, workplaces and all but essential business, mandatory stay-at-home policies, travel restrictions, border closures and others) have been deployed. Their urgent deployment was defended with modelling and observational data of spurious credibility. There is major debate on whether these measures are effective and there is also uncertainty about the magnitude of the harms that these measures might induce. Given that there is equipoise for how, when and if specific social distancing interventions for COVID-19 should be applied and removed/modified during reopening, we argue that informative randomised-controlled trials are needed. Only a few such randomised trials have already been conducted, but the ones done to-date demonstrate that a randomised trials agenda is feasible. We discuss here issues of study design choice, selection of comparators (intervention and controls), choice of outcomes and additional considerations for the conduct of such trials. We also discuss and refute common counter-arguments against the conduct of such trials.

The conduct of randomised-controlled trials (RCTs) is linked to the principle of equipoise, which assumes there is a conflict or uncertainty within the scientific community about the ‘relative therapeutic, prophylactic, or diagnostic merits of a set of interventions’ (London, 2017). Severe acute respiratory syndrome-coronavirus-2 (SARS-CoV-2) is a new and still poorly understood pathogen and in the absence of definitive pharmacologic treatments, non-pharmacological interventions (NPIs) have taken centre stage in the pandemic. These include measures collectively labelled social distancing, such as public gathering bans, closures of schools, workplaces and all but essential business, mandatory stay-at-home policies, travel restrictions, border closures and others. Here, we argue that RCTs are essential to perform in order to generate a reliable basis for major decisions pertaining to these NPIs. The existence of equipoise is an important condition for ethically acceptable research, because its presence indicates that studies are addressing a question of importance and in a way that does not knowingly make any participant worse off. RCTs of social distancing interventions are urgently needed. We offer examples of such trials and discuss issues of study design, choice of comparators, choice of outcomes, other considerations for the viable and informative conduct of these studies and address any residual counter-arguments against their conduct.

## Current status of suboptimal alternatives to RCTs: modelling and observational data

When the coronavirus disease 2019 (COVID-19) pandemic hit, no RCTs were available assessing any of these interventions even for other pathogens, e.g. influenza (Fong *et al*., 2020). Assessment of social distancing measures, including the most stringent ones like horizontal lockdowns, has been exclusively derived from observational (Pan *et al*., 2020) or modelling (Flaxman *et al*., 2020; Lai *et al*., 2020) studies on the current pandemic, and historical analysis of other epidemics (Markel *et al*., 2007). These types of evidence are useful but leave much uncertainty (Fong *et al*., 2020) and their inferences may be spurious.

Modelling studies are constrained to few alternatives and outcomes and hinge on the estimations of key parameters, which can be inaccurate or change over time. A modelling analysis (Flaxman *et al*., 2020) estimated 3.1 million deaths averted across 11 European countries due to NPIs and lockdown in particular. However, it is impossible to prove this huge benefit and disentangle how much is directly attributed to the implemented interventions and how much to other factors, like the accuracy of the prediction regarding number of deaths in the no intervention scenario. Other analyses of the same data have suggested that lockdown may have saved no lives (Chin *et al*., 2020). Moreover, even if the benefit was real, it is difficult to tell which among many measures in the bundle of ‘lockdown’ had the maximum benefits, and which might have had no benefits or even net harms.

Untoward consequences are rarely included, if at all, in such modelling calculations. For COVID-19, examples of harmful consequences of some social distancing interventions included increases in preventable deaths, as compulsory stay-at-home orders might make people wary of seeking medical help for other acute life-threatening problems (Solomon *et al*., 2020), or potential severe mental health consequences (Czeisler *et al*., 2020) and corresponding attributable excess mortality, owing to increases in domestic violence, unemployment, poverty or social isolation (Moser *et al*., 2020). Mid- and long-term harms may be even more substantial (Ioannidis, 2020).

Observational studies, particularly when historical or retrospective, are subject to confounding, selection bias and other biases, and again depend on accuracy and completeness of reporting. Historical analyses have questionable applicability outside of the particular past context. For instance, it is unclear if the experience with a different pathogen (e.g. 1918 influenza) can be transplanted to COVID-19?

For previous pandemics, the dearth of RCTs on social distancing could be defended because such extreme measures were rarely deployed at a global scale for prolonged periods of time. Moreover, in the last 75 years (when RCTs have become available) previous pandemics ended or were controlled before the need of obtaining more certain information about the effectiveness of adopted measures became an issue. Conversely, some modelling studies suggest that COVID-19 may persist into 2024, with prolonged/intermittent social distancing required into 2022 (Kissler *et al*., 2020). There is vast uncertainty about this claim, because it is still unknown whether ‘herd immunity’ may be reached earlier than anticipated, e.g. because of pre-existing cellular immunity (Grifoni *et al*., 2020) or because of lack of homogeneity in the mixing of populations (Gomes *et al*., 2020). However, the vast timescale, along with the global outreach of the pandemic, creates an imperative to obtain more reliable evidence about benefits and harms of different measures.

## RCTs of social distancing interventions conducted to-date in the COVID-19 era

Based on the ‘Living mapping and living systematic review of Covid-19 studies’ (https://covid-nma.com/the-project/), one RCT on social distancing measures in COVID-19 has been completed (Bretthauer, 2020), assessing whether gyms can be reopened. This study randomised 3764 individuals to two arms: allowing them to use training gym facilities (*n* = 1896) *v.* not using them (*n* = 1868). SARS-CoV-2 RT-PCR was done after two weeks and clinical assessment covered three weeks. None of the participants had outpatient visits or hospitalisations related to COVID-19 and only one tested positive for the virus, a participant randomised to the training arm but who had not used the facility at all.

Two additional randomised trials were brought to our attention through discussion with colleagues and their results have been released in the National Bureau of Economic Research and the SSRN preprint server, respectively (Banerjee *et al*., 2019; Angrist *et al*., 2020). One trial (Banerjee *et al*., 2019) conducted in India randomised people in West Bengal to a messaging campaign where a native Nobel laureate delivered messages *v.* messaging that referred to government information. Twenty-five million and three million individuals, respectively, were randomised to the intervention and control arms. The campaign was found to double the reporting of health symptoms to the community health workers, decrease travel beyond one's village, increase estimated handwashing, and probably the benefit spilled over to non-mentioned behaviours and also to non-recipients within the same community. The other trial (Angrist *et al*., 2020) randomised households in Botswana to three arms (SMS messaging, SMS messaging plus phone calls and control arm) and found that interventions improved the educational experience of students during lockdown.

These trials are imperfect, and they address focused questions, still leaving much to be desired. Moreover, one may wish to see trials addressing more fundamental questions, e.g. school opening rather than ways to improve education with closed schools. Nevertheless, their conduct suggests that the argument that one cannot do RCTs in an urgent situation, is refuted. These trials offer a proof-of-concept that RCTs are feasible.

## Resistance against RCTs in COVID-19 and confusing multiplicity of measures

Even if feasible, the conduct of RCTs on social distancing must overcome some substantial resistance of the status quo. Decision makers may not be familiar with or even scared of such designs and may have a defensive view to do anything provided they are not blamed of negligence. Getting buy-in for an RCT is essential for its implementation. A cluster-RCT of school districts with an ingenious rapid-cycle randomisation was proposed for assessing the effects of school closures, but there are no concrete implementation plans (Kolata, 2020). Another similar trial in Norway (http://www.isrctn.com/ISRCTN44152751) suspended recruitment because it could not accrue support from decision makers.

As the pandemic progresses, there is more measured fatigue among both policy makers and the general public. Some social distancing measures may cause major harms, and policy makers need to become aware of these risks and the uncertainty surrounding them. The general public is also starting to see the potentially major repercussions of lockdown measures in their daily life, finances and health. Long-term prospects are uncertain but frightening. If properly explained and supported by scientists, RCTs can hopefully find support in the public argumentation. It requires mostly a sense of honesty: that there is much that we don't know. There is a lot at stake that can end badly unless we find out what the best action plan is.

There is a wide misconception that taking measures (any measures) is a good thing, and that, in principle, taking more measures is even better. Governments, ministries, counties, institutions, businesses, and other authorities have cumulatively issued zillions of measures, orders and practice recommendations during this pandemic, often changing them in rapid sequence and in ways that are confusing and sometimes even obviously self-contradicting. For example, until 29 June 2020 the state of Michigan had issued 132 executive orders and Colorado has issued 115, with each executive order including potentially tens and hundreds of measures and details thereof (https://ballotpedia.org/Executive_orders_issued_by_governors_and_state_agencies_in_response_to_the_coronavirus_(COVID-19)_pandemic,_2020).

Regardless, the lack of consideration of harms in the dominant narrative and messaging regarding social distancing measures is highly concerning. Scientists and experts have routinely communicated the necessity of implementing social distancing to deal with an extremely major threat. Communication overarchingly relied on exuding certainty and instilling fear, most probably under the assumption that by conveying uncertainty, individuals would not comply with the enforced measures. This approach might have been necessary in the critical point of the pandemic wave as hospitals in some regions risked of being overrun, so as to increase adherence to exceptional and costly restrictions. Predictions from mathematical models with highly uncertain early inputs claimed catastrophe (Ioannidis *et al*., 2020). On the long run, assertive communication (e.g. ‘staying home saves lives’, ‘flatten the curve’) stifled critical discussions and ostracised uncertainty and equipoise considerations. The fact that high-quality data on these measures is lacking and needs to be gathered with urgency has received little attention in communication with the general public. However, past the immediate crisis, it is difficult to hide the huge uncertainty about the best course of action regarding when and how to reopen. Obviously, multiple reasonable competing alternatives exist.

A highly visible example of multiplicity of measures and uncertainty (that should justify equipoise) is what to do with universities. The College Crisis Initiative (https://collegecrisis.shinyapps.io/dashboard/) lists and updates the re-opening plans of ~3000 colleges, community colleges and universities in the United States. As of 26 August 2020, for the fall 2020 out of 2958 institutions, 177 planned to operate fully online, 793 primarily online, 457 adopted hybrid approaches, 578 proposed to operate primarily in person, 73 fully in person, 714 had not yet decided and 166 had other recipes. Within each of these large bins, institutions in the same bin might still differ in essential aspects of how exactly they planned to operate. Clearly, this vast diversity proves that we just do not know what is best to do and there are thousands of opportunities for testing different approaches head-to-head under equipoise. The situation with plans for re-opening of schools is even more confusing than for universities.

## Study designs for RCTs of social distancing interventions

RCTs of social distancing measures may randomise either individuals or groups and clusters. They may cover a wide spectrum: from the highly explanatory designs, more akin to clean, lab experiments done under ‘artificial’, ideal conditions among volunteering participants; to pragmatic, population-wide studies of complex interventions that are more messy, but come closer to real-life circumstances.

Designs potentially suitable for population-wide measures, such as cluster randomised trials of parallel clusters or stepped-wedge randomised trials (Hemming *et al*., 2015), have long existed and applied in other fields, including HIV and other infectious diseases, cancer, service delivery, criminal justice and other. The problem is not the lack of suitable study designs but the inappropriate erosion of equipoise and the potential resistance from politicians and other decision makers who may feel uneasy to say that they are withholding an intervention from some people. Some randomised designs can relieve the pressure from these stakeholders, since the intervention may still be applied to all groups. For example, stepped-wedge designs allow introducing an intervention to different clusters at different time points. Eventually all clusters are exposed to the intervention. However, one can still obtain a measure of the treatment effect from a randomised comparison.

The exact choice of best design depends on features which may vary according to what interventions need to be assessed and at what setting. For example, for cluster trials one has to consider the magnitude of the intra-cluster correlation, the potential for confounding by time (some interventions having different effectiveness when introduced earlier *v.* later), and the extent to which a cluster intervention may still require measurements in individual participants and, if so, whether these measurements may be affected by lack of blinding and of allocation concealment.

## Questions asked

Table 1 shows a number of aspects that can be addressed by RCTs of social distancing. Most trials compare an intervention against an inactive control. Such trials may be feasible also for NPIs, although sham controls are often difficult or impossible. Doing nothing may also be difficult to select as control in charged circumstances. Conversely, questions of dosing and titration of the various interventions may be more acceptable. For each of the adopted measures, one can think of many grades and doses that range from least disruptive to highly disruptive deployments of interventions. The vast diversity that exists on how different measures have been implemented in different countries or locations shows that selection of dose has been highly haphazard and arbitrary. Moreover, there can be widely acceptable equipoise for comparisons of timing of implementation or weaning (e.g. early *v.* later) of combinations of measures; sequencing of measures; and how exactly different measures can be combined.

**Table 1.** Aspects of social distancing interventions that can be considered to choose randomised comparisons

Important aspects	Examples
Interventions and their dosing	Physical distance: 1 m (like in France or Italy), 1.5 m (like in Australia), 6 foot (like in US or UK), 3 , 6 m (maximum distance virus can reach upon forceful cough) Closures of spaces where people can meet (decide on whether complete closure or some form of function still allowed): schools (decide on which grades and levels of education and whether any level of physical teaching is allowed in parallel or not); workplace; places of worship; pools; gym; clubs; restaurants; bars; shops (which ones); outdoor areas (e.g. parks, beaches); mass transit; non-essential businesses and services (define how is ‘non-essential’ defined) Cancellation of events where many people could meet: decide on types of events (e.g. movie theatres, music events, carnivals, conferences); decide on limit of people allowed (<10, <50, <1000) Travel restrictions: airport screening or other test before travelling; ban on travel between countries (define which); ban on travel within countries (define range of movement allowed) Quarantine (single person, contacts, large groups, whole buildings, whole towns, whole provinces; cordon sanitaire; define duration and how strictly enforced) Lockdown (all non-essential activities prohibited; outdoor activity permitted; interaction limited to few, repeated social contacts, e.g. ‘lockdown buddy’) Contact tracing (human *v.* digital; feasibility and usability of digital tools like mobile apps and tracking devices, e.g. electronic bracelets; adherence rates; voluntary *v.* mandated use) Screening: at building entry, at work, at other facilities (define type, sampling and frequency of screening)
Timing and duration of implementation	Pre-emptively (before any cases have been detected), early in the course of the epidemic (define how early and how timing is decided, based on what features or measurements), late in the course of the epidemic (similarly define) Duration (fixed, until the number of cases/reproductive rate/hospital or ICU occupation reaches a certain threshold)
Sequencing and combination	How different interventions at different doses are sequenced (in what order) and how they are combined
Enforcement of adherence	Advice, intense messaging (how intense and by which stakeholders, with what processes and with what means and media), law with fines, law with strong penalties, law with imprisonment, law with capital punishment
Weaning process	Refers to all of the items discussed above (interventions and dosing, timing, sequencing and combination, enforcement of adherence) but in reverse order, trying to remove restrictions
Measurement of extent of adoption	No measurement, tracking of indicators (decide which indicators)
Use of response indicators/surrogates	Using or not tracking indicators (e.g. number of documented infections, hospitalisations or deaths) to modulate the interventions, add more interventions, remove interventions
Target group choice	Entire population, specific groups defined by age, occupation/workplace, perceived risk factors (e.g. underlying predisposing diseases), or setting (see below)
Setting choice	World-wide, country-wide, town-wide, facility-wide (e.g. nursing homes, hospitals), more precisely localised (if so, decide based on which features)

Importantly, measures are theoretical constructs and what matters eventually is whether they are adhered to or not. A very aggressive measure may end up decreasing opportunities of infection to a lesser degree than a measure that is less aggressive, if the less aggressive measure may end up being easier to adopt and adhere to. For example, if students are deprived of direct participation in academic life (e.g. interaction with peers or mentors, use of libraries and study rooms, extra-curricular activities), and are constrained to move back home, they may unavoidably find other, perhaps even unsafe, opportunities for socialisation. How exactly adherence is enforced is also worth evaluating in RCTs. There is precedent of RCTs on interventions to improve adherence on other health problems (Kanters *et al*., 2017; Wiecek *et al*., 2019), for instance testing advice and messaging strategies (Thakkar *et al*., 2016). There is also literature on RCTs for using fines and penalties (Blæhr *et al*., 2018). These RCT designs can be transferred to the COVID-19 paradigm.

Given that most locations around the world have already adopted various social distancing measures, most of the RCTs done currently could focus more on the weaning of measures rather than their original adoption, but they can also examine questions surrounding their potential re-introduction. For example, a recent study proposed three network-based social distancing strategies designed to mitigate negative effects of social isolation (Block *et al*., 2020). These could be combined with health messaging strategies and tested in an RCT, if the need to increase social distancing in a region becomes stringent. Or the recently proposed ‘precautionary break’ lockdown combining very stringent NPI could be compared with the containment measures ‘as usual’ that are already applied. A modelling study (Keeling *et al*., 2020) appears to show this intervention offers an important ‘brake’ when infection rates are on the rise, potentially allowing other measures (e.g. contact tracing) to be reinstated. Given the substantial economic, societal and psychological costs, it would be useful to evaluate the relative efficacy of such a drastic measure.

RCTs can also address whether any indicators need to be tracked to see if measures are adopted or not, and whether feedback indicators should modulate the packaging and delivery of measures. Such trials can learn from trials that use adaptive designs guided by information on molecular and other markers in cancer and other fields (Korn and Freidlin, 2017).

Finally, RCTs may help identifying the optimal target group and setting where different social distancing measures should be implemented. We have substantial epidemiological knowledge already that the risk of serious outcomes and death with COVID-19 has a very steep age gradient (Davies *et al*., 2020; Levin *et al*., 2020), and there are strong risk factors reflected in predisposing diseases, and settings, such as specific facilities (e.g. nursing homes (Arons *et al*., 2020)). Trials could compare precise implementation of interventions in specific populations and settings *v.* wider population-level adoption, or may use risk information to choose the populations and settings where interventions are to be applied.

## Trial outcomes

Table 2 summarises considerations on choosing outcomes for social distancing interventions. Much like in other fields of medicine and public health, surrogate outcomes are easier to measure, but efforts should be made to measure outcomes that matter. A core outcome set for large-scale interventions COVID-19 trials is worthwhile developing, as has been done in multiple medical fields (e.g. the Core Outcome Measures in Effectiveness Trials initiative http://www.comet-initiative.org/). Documented COVID-19 infections are straightforward to capture, but even for this outcome, trialists should decide how it will be measured, e.g. with passive data collection or with aggressive testing. Issues of pragmatism also need to be considered (Loudon *et al*., 2015), because intense laboratory documentation of outcomes may not reflect the real world. Some trials with explanatory designs may nevertheless offer interesting hints about pathophysiology, e.g. transmission from asymptomatic patients or young children. Pragmatic trials, conversely, should focus on major clinical outcomes, e.g. hospitalisation, ICU admission and death.

**Table 2.** Key features to consider for outcomes for randomised trials of social distancing

Feature	Comment
Types of outcomes	Documented infections, hospitalisations or ICU hospitalisation for COVID-19, COVID-19 deaths, all deaths, quality of life indicators, impact on other diseases, impact on health utilisation, impact on social indicators (e.g. violence, unemployment)
Timing of measurement	Short-term, longer-term, determining carry-over effects and interactive effects from use of other interventions in parallel or in sequence
Data collection on outcomes	Passive, active, systematic, at different levels of intensity
Long-term relevance	Influence of short-term outcomes on longer-term prospects (e.g. few infections in the short term, but creating conditions for many more in the long term)
Cost-effectiveness	Strategies sustainable at short-, medium- and longer term; incorporation of cost-effectiveness metrics
Placing in context to broader gains and risks	From effects on economy, environment, society and health system at large

It is important to avoid having RCTs focus unilaterally on COVID-19 transmission and health consequences alone. Many measures, especially the most aggressive ones, could be very disruptive to many aspects of life, health and other dimensions. Measuring these additional dimensions of impact would enhance understanding their risk−benefit ratio. These include quality of life indicators, educational attainment, impact on other diseases, impact on health utilisation and impact on social indicators (e.g. violence, unemployment). Capturing these outcomes may require linking different types of databases.

RCTs can also experiment with different timing of measurements and short-term or long-term time horizons for assessing the final endpoints. Carry-over effects from sequential treatments, and interactions can be studied using the knowledge we have accrued on cross-over, adaptive and factorial designs from RCT applications in other fields. Moreover, dealing with COVID-19 is more likely to be a marathon than a sprint and therefore, long-term impact is more important than short-term outcomes. Aggressive lockdown measures may have better outcomes on infection rate in the short term, but if they lead to intervention fatigue and adverse effects on other aspects of health, economy or society, they may end up having a more unfavourable outcome profile than less aggressive interventions. Capturing the big picture of diverse outcomes is most informative.

## Rebuttal of additional counter-arguments

Many of the envisioned RCTs of social distancing may use cluster designs and they may not seek informed consent from individual participants. The lack of obtaining classic informed consent in cluster RCTs has already drawn attention and discussion on trials done for other topics in low-income countries. However, there is consensus that the conduct of such trials is entirely ethical, provided that equipoise exists (Weijer *et al*., 2012; MacKay and Chakrabarti, 2018; World Health Organization, 2019). Moreover, one should not forget that all the NPIs on COVID-19, including the most draconian ones, were imposed by governments and other policy makers without any consent of the targeted populations even though they may have placed high risk on the lives and livelihood of the participants.

Some scientists have argued that RCTs are not better, and are actually worse than observational data, for decision making (Deaton and Cartwright, 2018; Horwitz and Singer, 2018). However, this position has received critical counterarguments in the past (Ioannidis, 2018). This viewpoint has taken an extreme form in the writings, op-eds and social media activity of some militant researchers/activists during the COVID-19 pandemic. For instance, Trisha Greenhalgh, though supposedly an early advocate of evidence-based medicine, has even prophesised that the COVID-19 pandemic will largely be the end of evidence-based medicine and of the need for RCTs (Greenhalgh, 2020). We believe these trenchant positions are premised on a ‘strawman’ argument, which depicts advocating for gathering evidence with RCTs as the equivalent of implying that we do nothing until that evidence is collected and evaluated. To our knowledge, this argument has never been made. We agree that in many circumstances, particularly related to population-wide interventions and health, we cannot wait for evaluating the evidence *before* we take any action. We do not exclude the importance of natural experiments and a ‘practice-based evidence’ (Ogilvie *et al*., 2020). In truth, this has generally been the way in which NPI were evaluated in previous epidemics, when their deployment was more limited in time, scope and outreach.

Rather, acknowledging that COVID-19 is a protracted and global problem and that a multitude of policy actions to contain it are being deployed and modified in real time, we view RCTs are a way of reducing uncertainty about the chains of effects produced by these actions. Reducing uncertainty is particularly relevant when governments are scrambling with finding the ‘right’ combination and ‘dosing’ of containment strategies that would also minimise economic and societal harms. Moreover, RCTs provide intent-to-treat estimations, particularly relevant for interventions where adherence could be problematic or where the post-randomisation experiences of compared groups (e.g. one intervention could introduce several associated secondary changes) (Ioannidis, 2018) could be very different. This has significant pragmatic importance as many containment strategies target difficult to enforce changes in individual behaviours (e.g. government recommendations about the maximum number of people outside of the household that can be invited in private homes).

More broadly, the relative merits of different study designs need continuous evaluation and re-evaluation. RCTs and observational data can have different complementary contributions to generating meaningful evidence in different contexts (Ogilvie *et al*., 2020). Entirely eliminating a study design (actually the study design that inherently carries fewer problems with internal validity) seem to be an extreme position and may be particularly detrimental to science and public health.

A more cogent counter-argument is that although equipoise is a necessary condition for initiating a trial, it must also be the case that research has a strong chance of reducing that uncertainty and changing clinical practice. One may argue that RCTs would not be able to generate adequate evidence to satisfy this condition. We should acknowledge that most studies, both RCTs and others, indeed have limited usefulness (Ioannidis, 2016). However, this is not an issue limited to COVID-19 or to RCTs specifically. In fact, COVID-19 offers an opportunity to use what we have learned from research in other fields and to design trials that have a maximum chance of being useful and informative. This has been done multiple times in the past, and there is no reason why it cannot be done also in the COVID-19 setting. Nihilism should not prevail in thinking about an informative research agenda.

## Conclusion

Widespread, prolonged implementation of social distancing can have considerable individual and societal harms (Melnick and Ioannidis, 2020). If the benefit−harm ratio is uncertain, equipoise is upheld and should be communicated, and RCTs should be performed. Other scientists have also started examining the concept of performing RCTs for questions related to the effectiveness or implementation of social distancing policies (Haushofer and Metcalf, 2020; Starr, 2020). Further brainstorming should be encouraged.

Education of both scientists and the general public about the need of preserving and communicating equipoise is crucial. Particularly given the speed with which information is shared on social media, researchers need to understand the importance in communicating uncertainty. Advocacy can be extremely beneficial for societal causes, but detrimental when attached to interventions of uncertain harm−benefit ratio, as are some social distancing measures under some circumstances. Advocacy in this context can undermine equipoise and hamper the planning of RCTs.
